# Multi-Agent Cooperative Target Search

**DOI:** 10.3390/s140609408

**Published:** 2014-05-26

**Authors:** Jinwen Hu, Lihua Xie, Jun Xu, Zhao Xu

**Affiliations:** 1 Singapore Institute of Manufacturing Technology, Singapore 638075, Singapore; E-Mail: jwhu@simtech.a-star.edu.sg; 2 The School of Electrical and Electronic Engineering, Nanyang Technological University, Singapore 639798, Singapore; E-Mail: elhxie@ntu.edu.sg; 3 Western Digital Corporation, Singapore 118261, Singapore; E-Mail: jun.xu@wdc.com; 4 Institute of High Performance Computing, Singapore 138632, Singapore

**Keywords:** UAV, multi-agent network, target search, cooperative control

## Abstract

This paper addresses a vision-based cooperative search for multiple mobile ground targets by a group of unmanned aerial vehicles (UAVs) with limited sensing and communication capabilities. The airborne camera on each UAV has a limited field of view and its target discriminability varies as a function of altitude. First, by dividing the whole surveillance region into cells, a probability map can be formed for each UAV indicating the probability of target existence within each cell. Then, we propose a distributed probability map updating model which includes the fusion of measurement information, information sharing among neighboring agents, information decay and transmission due to environmental changes such as the target movement. Furthermore, we formulate the target search problem as a multi-agent cooperative coverage control problem by optimizing the collective coverage area and the detection performance. The proposed map updating model and the cooperative control scheme are distributed, *i.e.*, assuming that each agent only communicates with its neighbors within its communication range. Finally, the effectiveness of the proposed algorithms is illustrated by simulation.

## 1. Introduction

With the fast development of high resolution imaging devices and processing technologies, unmanned aerial vehicles (UAVs) with air-borne cameras are increasingly employed in civil and military applications such as environmental monitoring, battlefield surveillance and map building, where ground-target search is one of the major applications [1,2]. Target tracking and search have been one of the most popular utilizations of UAVs [3,4]. The conventional method for target search by UAVs in a closed region divides the whole surveillance region into cells, and associates each cell with a probability or confidence of target existence in the cell which constitutes a probability map for the whole region [5,6].

In [7], an online planning and control method is proposed for cooperative search by a group of UAVs, where each agent keeps an individual probability map for the whole region updated according to the Dempster-Shafer theory. A path planning algorithm is designed by using the obtained measurement information, which requires each agent to directly communicate with all other agents. In [8], target detection is considered as part of an integrated mission including coverage control and data collection as parallel tasks for multi-agent networks. The coverage control method aims to maximize the joint detection probability of random events and the probability of target existence is updated by measurements based on the Bayesian rule. However, only the measurement information of direct neighbors is exchanged, which makes it difficult to obtain the target information of the whole surveillance region. In [9], a decentralized gradient-based control strategy is proposed for multiple autonomous mobile sensor agents searching for targets of interest by minimizing the joint team probability of no detection within action horizon based on range detection sensing model. However, each agent is required to collect detection information from all other agents. In [10], a decentralized search algorithm is developed which includes a two-step updating procedure for the probability maps. Each agent first obtains observations over the cells within its sensing region and updates its individual probability map by the Bayesian rule. Then, each agent transmits its individual probability map to its neighbors for map fusion. This algorithm is distributed and full network connectivity is not required. However, the lack of information correlation makes the map fusion difficult and only a heuristic fusion method is given in [10], the performance of which has not been analyzed. In our recent work [11], a distributed iterative map updating model is proposed to fuse the information from measurements and the maps of neighbors based on a logarithmic transformation of the Bayesian rule. Through this, the nonlinear Bayesian update is replaced by a linear one which simplifies the computation. The convergence speed of individual probability map of an agent is also analyzed under fixed detection and false alarm probabilities for the search of static targets.

The cooperative control is an important task for efficient target search by a group of UAVs. Compared with the centralized control algorithms, distributed control algorithms are more robust to accidental failures of UAVs and breaks of communication links [12]. In [13], a distributed multi-agent coverage control method is proposed based on a given sensing performance function related to the distance to robots and gradient descent algorithms are designed for a class of utility functions to optimize the coverage and sensing performance. In [14], a distributed, adaptive control law is developed to achieve an optimal sensing configuration for a network of mobile robots which obtain sensory information of a static environment and exchange their estimates of the environment with neighbors. In [15], a three dimensional distributed control strategy is proposed to deploy hovering robots with downward facing cameras to collectively monitor an environment. A new optimization criterion is defined as the information obtained by each pixel of a camera. In [16], a dynamic awareness model is proposed to control a multi-vehicle sensor network with intermittent communications. The state of awareness of each individual vehicle is updated by its own sensing model and sharing information with its neighbors. However, none of the coverage control schemes mentioned above has considered the detection results of target existence which may affect UAVs' movement decisions in target search. Moreover, there are very few works addressing the issue of distributed vision-based cooperative search for multiple mobile targets with probabilistic detections.

In this paper, we investigate the vision-based cooperative search for multiple ground mobile targets by a group of UAVs with limited sensing and communication capabilities. The main contribution of this paper is that a distributed strategy of information fusion and cooperative control is proposed for searching multiple mobile targets using multi-agent networks based on probabilistic detections. In addition, the time-varying detection and false alarm probabilities are considered which are due to the varying altitudes of the agents with 3-dimensional dynamics. Each agent under our search strategy shares local target information and controls its own behavior in a distributed manner. Based on the probability map updating model proposed in [11], we generalize the model by considering the information decay and transmission between cells due to environmental changes such as the target movement. The influence of the time-varying detection probability on the update of probability maps due to the three-dimensional UAV dynamics is also analyzed. Then, a coverage optimization problem is formulated to balance the coverage area and detection performance. The proposed map updating model and cooperative control scheme are distributed, *i.e.*, each agent only communicates with the agents within its communication range.

This paper is organized as follows: Section 2 describes the basic notations and assumptions used in this paper. Section 3 presents the probability map updates by measurements and information sharing with time-varying detection probabilities. In Section 4, a three-dimensional coverage control method is presented for target search. Simulation results are shown in Section 5, and the conclusions are drawn in Section 6.

## 2. Basic Definitions and Assumptions

**Figure 1.**
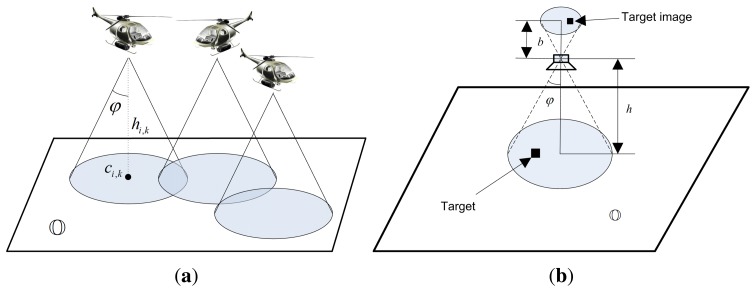
Target search by multiple UAVs. (**a**) A network of UAVs; (**b**) Target image taken by an airborne camera.

The surveillance region 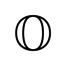 ∈ ℝ^2^ is assumed to be on a plane ground and has been uniformly divided into a set of cells of the same size. We assume that all UAVs (or agents) use the same global Cartesian coordinate system and the position of each agent is denoted as $\mu_{i,k}={\left[c_{i,k}^{T},h_{i,k}\right]}^{T}$ ∈ ℝ^3^ for agent *i* (*i* = 1, 2, ⋯, *N*) at time *k* (as shown in Figure 1a), where *c _i,k_* ∈ ℝ^2^ is the planar coordinate of its projection on 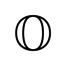, *h_i,k_* ∈ ℝ is the altitude of the agent above 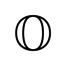, *N* is the total number of agents and “T” denotes the transpose operation. Each agent is assumed to have access to its own position at any time. Each cell in the surveillance region is associated with a probability or confidence of target existence within the cell which is modeled using the Bernoulli distribution, *i.e., θ_g_,_k_* = 1 (a target is present) with probability *P_i_* (*θ_g,k_* = 1) and *θ_g,k_* = 0 (no target is present) with probability 1 − *P_i_* (*θ_g_* = 1) for agent *i* and cell *g* at time *k*, where *g* ∈ ℝ^2^ is the location of the cell center in 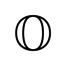. If more than one target are present within a cell, they are treated as one single target.

In this paper, we mainly discuss about the vision-based detections where each agent carries an airborne camera facing downward to surveillance region (as shown in Figure 1a). Each agent independently takes measurements *Z_i,g,k_* over the cells within its sensing region ℂ*_i,k_* at time *k*, where

$${\mathbb{C}}_{i,k}≜\{g\in \text{O}:\left\|g-c_{i,k}\right\|⩽h_{i,k}\text{tan}\varphi \}$$

and ‖•‖ denotes the 2-norm for vectors. Each agent is assumed to have the same angle of field of view, half of which is denoted by *φ*. We also assume that the size of each cell is sufficiently small comparing with the size of ℂ*_i,k_* so that we can ignore the boundary effect and roughly consider a cell as wholly within ℂ*_i,k_* if its center is within ℂ*_i,k_*. Only two observation results are defined for each cell, *Z_i,g,k_* = 0 or *Z_i,g,k_* = 1. For all cells, *P* (*Z_i,g,k_* = 1|*θ_g,k_* = 1) = *p_i,k_* and *P* (*Z_i,g,k_*= 1|*θ_g,k_* = 0) = *q_i,k_* are assumed to be known by agent *i* as the detection probability and false alarm probability respectively.

The topology of the network of all agents at time *k* is modeled by an undirected graph 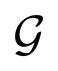 *_k_* = (*ε_k_*, 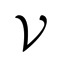). 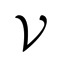 = {1,2,…,*N*} is the vertex set and *ε_k_* ={{*i,j*} : *i,j* ∈ 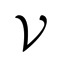; ‖*μ_i,k_*− *μ_j,k_*‖ ⩽ *R_c_*} is the edge set, where each edge {*i, j*} is an unordered pair of distinct agents and *R_c_* is the communication range of each agent. The graph or the network is connected if for any two vertices *i* and *j* there exists a sequence of edges (a path) {*i, ν*_1_}, {*ν*_1_, *ν*_2_},…, {*ν_n_*_−1_, *ν_n_*}, {*ν_n_*,*j*} in *ε_k_*. Let 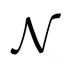 *_i,k_* = {*j* ∈ 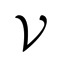
| {*i,j*} ∈ *ε_k_*} U {*i*} denote the set of neighbors of agent *i* at time *k* where an agent is assumed to be a neighbor of itself. The degree (number of neighbors) of agent *i* at time *k* is denoted as *d_i,k_* = | 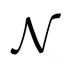 *_i,k_*|.

## 3. Probability Map Update

### 3.1. Bayesian Update and Consensus-Based Map Fusion

In [11], we proposed a cooperative control scheme for target search in multi-agent systems. In a group of UAVs, each agent *i* keeps an individual probability map 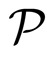 *_i_*,*_g,k_* of the whole region, where 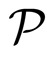 *_i,g,k_* ≜ *P_i_* (*θ_g,k_* = 1) and is updated by the Bayesain rule:

(1) $$\begin{array}{ll} P_{i,g,k} & =\frac{P(Z_{i,g,k}|\theta_{g,k}=1)P_{i,g,k-1}}{P(Z_{i,g,k}|\theta_{g,k}=1)P_{i,g,k-1}+P(Z_{i,g,k}|\theta_{g,k}=0)(1-P_{i,g,k-1})}\\ & =\{\begin{array}{ll} \frac{p_{i,k}P_{i,g,k-1}}{p_{i,k}P_{i,g,k-1}+q_{i,k}(1-P_{i,g,k-1})} & \text{if}\;Z_{i,g,k}=1\\ \frac{(1-p_{i,k})P_{i,g,k-1}}{\left(1-p_{i,k})P_{i,g,k-1}+(1-q_{i,k})(1-P_{i,g,k-1}\right)} & \text{if}\;Z_{i,g,k}=0\\ P_{i,g,k-1} & \text{ohterwise} \end{array} \end{array}$$

where 0 < 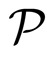 *_i,g_*,_0_ < 1 and 1 > *p_i,k_*,*q_i,k_* > 0. For the cases with *p_i,k_* = 0 or 1 or *q_i,k_* = 0 or 1, simplified conclusions can be obtained as shown in [11] and will not be considered in this paper. By letting

(2) $$Q_{i,g,k}=\ln\left(\frac{1}{P_{i,g,k}}-1\right)$$

we get the following transformation of Equation (1):

(3) $$Q_{i,g,k}=Q_{i,g,k-1}+\upsilon_{i,g,k}$$

where

(4) $$\upsilon_{i,g,k}≜\{\begin{array}{ll} \ln\frac{q_{i,k}}{p_{i,k}} & \text{if}\;Z_{i,g,k}=1\\ \ln\frac{1-q_{i,k}}{1-p_{i,k}} & \text{if}\;Z_{i,g,k}=0\\ 0 & \text{otherwise} \end{array}$$

Keeping *Q_i,g,k_* as the updated term instead of 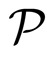 *_i,g,k_* simplifies the nonlinear update in Equation (1) into the linear one in Equation (3). For a group of UAVs, we let each agent *i* at time *k* first take measurements and transmit the measurements to its neighbors. After receiving the measurements from all its neighbors, *Q_i,g,k_* is updated as follows:

(5) $$H_{i,g,k}=Q_{i,g,k-1}+\underset{j\in N_{i,k}}{\text{∑}}\upsilon_{j,g,k}$$

Then, each agent *i* transmits the updated *Q_i,g,k_* of the whole region to its neighbors for map fusion, which is given by:

(6) $$Q_{i,g,k}=\underset{j\in N_{i,k}}{\text{∑}}w_{i,j,k}H_{j,g,k}$$

where $w_{i,i,k}=1-\frac{d_{i,k}-1}{N}$, $w_{i,j,k}=\frac{1}{N}$ for *j* ∈ 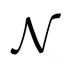 *_i,k_* (*j* ≠ *i*) and *w_i,j,k_*= 0 for *j* ∉ 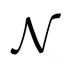 *_i,k_*. Then, a matrix composed of *w_i,j,k_* can be defined as:

(7) $$W_{k}≜{\left[w_{i,j,k}\right]}_{N\times N}(i,j=1,\ldots ,N)$$

which is a doubly stochastic matrix [17]. The communications of neighboring agents are assumed to be synchronized within a short time interval. Time synchronization in distributed networks is not the focus of this paper and has been addressed by many works [18–21].

### 3.2. Time-Varying Detection Probability

In [11], we only considered a 2-dimensional control scheme assuming that all agents move on a fixed plane parallel to the ground plane. However, in the real world, UAVs such as helicopters can change their altitudes according to their task requirements so as to enlarge their sensing area (here we only consider cameras with a fixed zooming level). Therefore, in this paper, we will consider the influence of 3-dimensional dynamics of UAVs on the detection performance.

For vision-based detection, the detection probability relies on the picture resolutions. Figure 1b shows the basic imaging scheme by an airborne camera similar to the one given in [15,22]. In general, a desirable property for good target recognition is a “right” ratio between the size of the image and the size of the target, where “right” depends on the target type and the detection algorithm that is employed. To be more simplified, it can be assumed that the larger the image of a target in the picture (in terms of the number of occupied pixels) obtained by the UAV, the easier for the UAV to discriminate the target no matter what recognition method is used. Hence, we can model the target discriminability of a UAV as a function *ρ* proportional to the ratio between the size of a target image taken by the camera denoted by *S*_TI_ and the size of one pixel denoted by *S*_P_, *i.e.*, $\rho \propto \frac{S_{\text{TI}}}{S_{\text{P}}}$. Here, we assume that all targets are of the same visual properties such as color, shape and size that are influential on target discriminability. It is also assumed that each camera has a fixed focal length so that we can only consider the change of *ρ* due to the variation of agent altitude. Then, by denoting the size of the projection of a target on the ground plane as *S*_T_, we can derive that:

(8) $$\rho \propto \frac{S_{\text{TI}}}{S_{\text{T}}}\frac{S_{\text{T}}}{S_{\text{P}}}=\frac{b^{2}S_{\text{T}}}{h^{2}S_{\text{P}}}$$

where *h* is the altitude of the UAV and *b* is the fixed distance between the image and the lens (as shown in Figure 1b). In a multi-agent system, for the *i*-th agent at time *k*, we have $\rho_{i,k}\propto \frac{b^{2}S_{\text{T}}}{h_{i,k}^{2}S_{\text{P}}}$. From Equation (8), we may get *ρ_i,k_* → ∞ as *h_i,k_* → 0. However, in reality, *ρ_i,k_* cannot be infinitely large and there should be an upper limit when *h_i,k_* is smaller than a threshold *h̲*. That is to say, the target discrimination ability will not be improved any more if a UAV is descending very close to the ground.

The target discriminability determines the detection probability when a UAV is detecting the existence of targets within each cell under surveillance. It is natural to conceive that the detection probability *p_i,k_* increases and the false alarm probability *q_i,k_*decreases as *ρ_i,k_* increases. When the altitude of the UAV becomes larger than a threshold *h̄*, it runs out of its ability to discriminate any target from the background environment, which means that the detection result dose not rely on the true existence of the target any more. That is, if *h_i,k_* ⩾ *h̄*, we have *P* (*Z_i,g_*,*_k_* = 0|*θ_g_* = 1) = *P* (*Z_i,g,k_* = 1|*θ_g_* = 1) = *P* (*Z_i,g,k_* = 1|*θ_g_* = 0) = *P* (*Z_i,g,k_* = 1|*θ_g_* = 0), *i.e., p_i,k_* = *q_i,k_* = 0.5. Generally, when *h̲_i,k_* ∈ [*h*, *h̄*] (*h̲*<*h̄*), *p_i,k_* should be a monotonically increasing function of *ρ_i,k_*, or more explicitly, a monotonically decreasing function of *h_i,k_*. Therefore, we assume the following detection probability model:

(9) $$p_{i,k}=\{\begin{array}{ll} 0.5 & \text{if}\;h_{i,k}⩾\bar{h}\\ f_{1}(h_{i,k}) & \text{if}\;\underline{h}<h_{i,k}<\bar{h}\\ 0 & \text{if}\;0<h_{i,k}⩽\underline{h} \end{array}$$

where $f_{1}^{\prime }(h_{i,k})<0$ for *h_i,k_* ∈ (*h̲, h̄*) and 1 > *f*_1_ (*h̲*) = *p̆* > *f*_1_ (*h̄*) = 0.5. Similarly, we can assume the false alarm probability model as a monotonically increasing function of *h_i,k_*:

(10) $$q_{i,k}=\{\begin{array}{ll} 0.5 & \text{if}\;h_{i,k}⩾\bar{h}\\ f_{2}(h_{i,k}) & \text{if}\;\underline{h}<h_{i,k}<\bar{h}\\ 0 & \text{if}\;0<h_{i,k}⩽\underline{h} \end{array}$$

where $f_{2}^{\prime }(h_{i,k})>0$ for *h_i,k_* ∈ (*h̲, h̄*), 0 < *f*_2_ (*h̲*) = *qˆ* < *f*_2_ (*h̄*) = 0.5. In this paper, the altitude *h_i,k_*of an agent is allowed to vary from 0 to ∞ for theoretic analysis, though it may not happen in the real world due to system limitations.

#### Remark 1

*Model* (9) *is motivated by the natural understanding of the interaction between the altitude of an agent and its detection and false alarm probabilities. It only reflects the general relation between those parameters, and is not restricted to a specific parametric representation of f*_1_ *and f*_2_*. Hence, our method is applicable for any detection probability function that fits for the model. An experimental detection probability model of CCD camera has been given in* [22].

#### Remark 2

*p_i,k_ and q_i,k_ can also be cell-dependent, i.e., they may vary from place to place due to environment conditions. For example, the target is often easier to be discriminated on an open ground than on a land with trees. In complex environments, agents must know the detection probability and false alarm probability models of different type of regions. For the ease of expression, we assume the models to be constant across the whole surveillance region*.

Denoting by *m_i,g,k_* the number of observations taken over cell *g* up to time *k* by agent *i* and defining $m_{g,k={\text{∑}}_{i=1}^{N}m_{i,g,k}}$, we can get the following conclusions for the update of *Q_i,g,k_*.

#### Theorem 1

*Given the initial prior probability map* 0 < 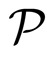 *_i,g_*,_0_ < 1 ∀*_i_* ∈ 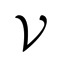, *if there exists a constant ε* > 0 *such that p_i,k_* ⩾ 0.5 + *ε and q_i,k_* ⩽ 0.5 − *ε*∀*i* ∈ 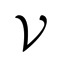, *and the network topology* 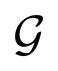 *_k_ is connected at all times, the following conclusions hold by implementing the map updating rule* (5) *and* (6).

(1) If a target is present within cell *g*, $Q_{i,g,k}\overset{a.s.}{\to }-\infty$ (*i.e.*, $P_{i,g,k}\overset{a.s.}{\to }1$) ∀*i* ∈ 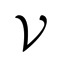 as *m_g,k_* → +∞.
(2) If no target is present within cell *g*, $Q_{i,g,k}\overset{a.s.}{\to }+\infty$ (i.e., $P_{i,g,k}\overset{a.s.}{\to }0$) ∀*i* ∈ 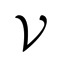 as *m_g,k_* → +∞.

#### Proof

See Appendix A.

### 3.3. Environment-Based Probability Map

In the map updates (5) and (6), the effect of the environmental changes such as the information decay and transmission between cells has not been considered. For example, if targets may randomly appear or disappear during search, the historical information about the target existence cannot reflect the true current situation and revisits of certain frequency to the detected cells are needed for information update. This problem can be formulated as the information decay for each cell. If a target may move from one cell to another, then part of the information for the former cell should be removed and counted as the new information for the latter cell. This problem can be formulated as the information transmission between each two cells. Therefore, we need to generalize the aforementioned map updating model to be applicable to the case with such environmental changes. Similar to the assumption made in [16], we assume that *Q_i_*,*_g,k_* decays exponentially for each cell if there is no prior knowledge and/or no measurement information. The information transmission between cells due to target movement is modeled based on the transition of probabilities. In addition, the prior knowledge about the environmental change is taken as the system input. All these lead to the following generalized updating model for *Q_i,g,k_*:

(11) $$\begin{array}{ll} H_{i,g,k} & =e^{-\alpha T}\underset{r\in \text{O}}{\text{∑}}a_{i,g,r,k}b_{i,g,r,k}Q_{i,r,k-1}+\underset{j\in N_{i,k}}{\text{∑}}\upsilon_{j,g,k}+\xi_{i,g,k}\\ Q_{i,g,k} & =\underset{j\in N_{i,k}}{\text{∑}}w_{i,j,k}H_{j,g,k} \end{array}$$

where *α* ⩾ 0 is the information decaying factor, *T* is the sampling period of all UAVs, *a_i,g,r,k_*and *b_i,g,r,k_* are the information transmission factors which are nonnegative, and *ξ_i,g,k_* is the input information vector given by the prior knowledge about the target existence within cell *g*. Specifically, *b_i,g,r,k_* satisfies *b_i,g,g,k_* = 1 and *b_i,g,r,k_* = 0 (*g* ≠ *r*) for *Q_i,r,k_*_−1_ > 0, and *b_i,g,r,k_* = *P*(*θ_g,k_* = 1|*θ_r,k_*_−1_ = 1) for *Q_i,r,k_*_−1_ ⩽ 0. *a_i,g,r,k_* is determined by *a_i_*,*_g_*,*_rˆi,k_* = 1 and *a_i,g,r,k_* = 0(*r* ≠ *rˆ_i_*), where

(12) $$\begin{array}{ll} {\hat{r}}_{i} & =\underset{r\in B_{i,g,k}}{\text{argmin}\;}b_{i,g,r,k}Q_{i,r,k-1}\\ B_{i,g,k} & =\left\{r\in \text{O}:b_{i,g,r,k}>0\right\} \end{array}$$

#### Remark 3

*a_i,g,r,k_ and b_i,g,r,k_ are defined based on the physical meaning of information transmission due to the target movement in the real world. Since the combination of Q_i,r,k_* (*r* ∈ 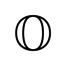) *into a cell g involves the combination of historical measurement information of all cells r* ∈ 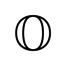 , *the correlation of which may not be known, we need to be careful in dealing with the fusion of such information. If Q_i,g,k_* > 0, *we are more confident that no target exists within cell g. Otherwise, we are more confident that a target exists within cell g. Since an information transmission out of a cell at time k is expected to occur only when a target exists within the cell at time k*−1, *we let b_i,g,r,k_* = 0 *if Q_i,r,k_*_−1_ > 0, *which means there is no transmission of information* (*or target movement*) *from cell r to cell g. If Q_i,r,k_*_−1_ ⩽ 0, *an information transmission occurs from cell r to cell g due to the possible target movement from r to g and the amount of information transmitted should be proportional to P* (*θ_g,k_* = 1|*θ_r,k_*_−1_ = 1), *i.e., equal to b_i,g,r,k_Q_i,r,k_*_−1_*. The smaller b_i,g,r,k_ is, the less amount of information is retained for cell g. Furthermore, by assuming that within one cell there can only exist up to one target at a time, i.e., at most one target can move into a cell at a time, we select the information stream with the largest transmitted amount as the newly stored information for cell g when there are incoming information streams from multiple cells r* ∈ 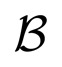 *_g,k_. That is, to take the smallest b_i,g,r,k_Q_i,r,k_*_−1_ *subject to Q_i,r,k_*_−1_ ⩽ 0 *as the newly stored information after the transmission, which corresponds to the most probable target movement to g in all possible movements to g from different cells. The information decaying factor α is set to be positive in the case that the prior knowledge of b_i,g,r,k_ is not accurate or targets may appear and disappear unpredictably during the search. In this case, the information decay makes the agents revisit the detected regions at a certain frequency. As for the input information vector ξ, it only denotes the effect brought by the prior knowledge and there is no need to calculate it out in real implementations, because any prior knowledge on the target existence can be directly used to update the probabilities of target existence and thus update directly following its definition in* Equation (2).

*Here we give a simple example to illustrate how the parameters are designed if the true target dynamic model is give by x _k_*_+1_ = Ψ*x_k_ where x_k_ is a vector including the target location. In this case, one can calculate the transition probability P*(*θ_g,k_*_+1_ = 1|*θ_r,k_*= 1) *for any two cells r and g where θ_r,k_* = 1 *represents that the target locates within cell r at time k. Following this, given the current accumulated information on target existence Q_i,r,k_ of agent ifor cell r at time k, one can calculate b_i,g,r,k_ following its definition. Further, with the results of b_i,g,r,k_ for any two cells r and g, one can calculate a_i,g,r,k_ according to its definition in* Equation (12). *If the target will not suddenly disappear/appear, the decaying factor α can be set as zero*.

Define the following augmented variables:

$$\begin{array}{ll} Q_{i,k} & ={\left[Q_{i,g_{1},k,\ldots ,Q_{i,g_{M},k}}\right]}^{\text{T}},{\text{Q}}_{k}≜{\left[{\text{Q}}_{1,k}^{\text{T}},\ldots ,Q_{N,K}^{\text{T}}\right]}^{\text{T}}\\ V_{i,k} & ={\left[{\text{∑}}_{j\in N_{i,k}}\upsilon_{j,g_{1},k},\ldots ,{\text{∑}}_{j\in N_{i,k}}\upsilon_{j,g_{M},k}\right]}^{\text{T}}\\ V_{k} & ={\left[{\text{V}}_{1,k}^{\text{T}},\ldots ,{\text{V}}_{N,k}^{\text{T}}\right]}^{\text{T}}\\ \xi_{i,k} & \begin{array}{cc} ={\left[\xi_{i,g_{1},k},\ldots ,\xi_{i,g_{1},k}\right]}^{\text{T}}, & \xi_{k}={\left[\xi_{1,k}^{\text{T}},\ldots ,\xi_{N,k}^{\text{T}}\right]}^{\text{T}} \end{array}\\ A_{i,k} & ={\left[a_{i,g_{\tau },g_{s},k}b_{i,g_{\tau },g_{s},k}\right]}_{M\times M}(\tau ,s=1,\ldots ,M)\\ A_{k} & =\text{diag}\left[A_{1,k},\ldots ,A_{N,k}\right] \end{array}$$

where *τ* and *s* are respectively the row and column indices of an appropriate cell in *A_i,k_*, and *M* is the total number of cells, we get the following generalized updating model:

(13) $${\text{Q}}_{k}=e^{-\alpha T}(W_{k}⊗I)A_{k}{\text{Q}}_{k-1}+(W_{k}⊗I)({\text{V}}_{k}+\xi_{k})$$

where ⊗ denotes the Kronecker product.

According to Theorem 1, ‖**Q***_k_*‖ can be seen as the gathered information for decision making on the target existence and the larger the ‖**Q***_k_*‖, the less the uncertainty about the target existence or nonexistence. Hence, our aim of controlling the UAVs is to maximize ‖**Q***_k_*‖ in some sense, which will be discussed in the following section.

## 4. Cooperative Coverage Control

In the previous section, a distributed map updating scheme was proposed for fusion of the knowledge of multiple agents. In this section, we will design a cooperative control strategy that optimizes the trajectories of agents for target search based on their real-time updated knowledge about the target information. Within each time interval, an agent first updates its probability map by the the map updating scheme designed in Section 3.3 and then makes a control decision on which place it should move to for the next observation by collective optimization which will be addressed in this section. The two steps make the whole network form a closed-loop sensing and feedback control system.

Here we consider the waypoint motion model for each agent:

(14) $$\mu_{i,k}=\mu_{i,k-1}+u_{i,k}$$

where *u_i,k_* ∈ ℝ^3^ is the control input (or the waypoint displacement) of the *i*-th agent at time *k*. Note that the above motion model only deals with the waypoints of agents at discrete-time steps. The true dynamics of agents is not discussed in this paper since we do not want to limit our results on the dynamic model of any specific type of UAV. How to make the agents achieve the desired waypoints by their inner-loop flight controllers is a technical issue which will not be addressed in this paper but left to be solved in our real system experiments. Our job is to optimize the selection of the next waypoint (*i.e., u_i,k_*) for each agent given its current waypoint (*i.e., u_i,k_*_−1_).

Following Equation (13), we can get

$${\text{Q}}_{k}={\text{G}}_{k}+(W_{k}⊗I){\text{V}}_{k}$$

where **G***_k_* ≜ *e*^−^*^αT^* (*W_k_* ⊗ *I*) *A_k_***Q***_k_*_−1_+ (*W_k_* ⊗*I*) ***ξ****_k_*. At time *k* − 1, **G***_k_* can be seen as the prior information, and **V***_k_* the information gathered from measurements. Since **V***_k_* and E [**V***_k_*] are both related to the true target existence which is unknown, we cannot predict the values of **Q***_k_* or E [**Q***_k_*] before taking measurements. What we can do at time *k* − 1 is to find the optimal next time sampling position *μ_i,k_* so as to maximize the information to be gathered at time *k*. More precisely, the problem can be formulated as the optimization problem:

(15) $$\underset{\mu_{k}}{\text{max}\;}\text{E}\;\left[{\left\|{\text{Q}}_{k}-{\text{G}}_{k}\right\|}^{2}|Q_{k-1,}\xi_{k}\right]$$

where $\mu_{k}≜{\left[\mu_{1,k}^{\text{T}},\ldots ,\mu_{N,k}^{\text{T}}\right]}^{\text{T}}$. Considering that *W_k_* includes the global topological information which is often hard to obtain for each individual agent in a distributed system, and ‖(*W_k_* ≜ *I*) **V***_k_*‖ ⩽ ‖**V***_k_*‖, we replace Equation (15) with the following suboptimal optimization:

(16) $$\underset{\mu_{k}}{\max\;}\text{E}\;\left[{\left\|{\text{V}}_{k}\right\|}^{2}\right]=\underset{g\in \text{O}}{\text{∑}}\overset{N}{\underset{i=1}{\text{∑}}}\underset{j\in N_{i,k}}{\text{∑}}\text{E}\;\left[\upsilon_{j,g,k}^{2}\right]1_{\left\{g\in {\mathbb{C}}_{j,k}\right\}}$$

Notice that Equation (16) is not an approximation of Equation (15), but a new cost function we intend to optimize which is an upper bound of Equation (16). Such way of defining the cost function is very common in statistics and estimation theory such as the Cramér–Rao lower bound, which is often selected as the cost function if the true variance of estimation error is time-varying and unknown.

Following Equation (16), we should try to maximize $\text{E}\;\left[\upsilon_{j,g,k}^{2}\right]$ and the collective sensing area of all agents. From Equation (4), we get for *g* ∈ ℂ*_j,k_*:

$$\text{E}\;\left[\upsilon_{j,g,k}^{2}\right]=\{\begin{array}{ll} {\left\|p_{j,k}\ln\frac{q_{j,k}}{p_{j,k}}+(1-q_{j,k})\ln\frac{1-q_{j,k}}{1-p_{j,k}}\right\|}^{2} & \text{if}\;\theta_{g,k}=1\\ {\left\|q_{j,k}\ln\frac{q_{j,k}}{p_{j,k}}+(1-p_{j,k})\ln\frac{1-q_{j,k}}{1-p_{j,k}}\right\|}^{2} & \text{otherwise} \end{array}$$

It is straightforward to find that $\text{E}\;\left[\upsilon_{j,g,k}^{2}\right]$ is monotonically increasing with respect to *p_j,k_* and monotonically decreasing with respect to *q_j,k_* no matter *θ_g,k_* = 1 or not. Thus, Equation (16) is further replaced with the following optimization problem:

(17) $$\underset{\mu_{k}}{\max}H\;(\mu_{k})=\overset{N}{\underset{i=1}{\text{∑}}}\int_{{\text{M}}_{i,k}}\phi_{k}(r)(p_{i,k}-q_{i,k})1_{\left\{r\in {\mathbb{C}}_{i,k}\right\}}dr$$

where *ϕ_k_* is a given nonnegative weighting function of *r* ∈ 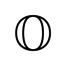 at time *k*, and its influence on the control law will be shown later. { 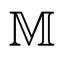
_1_,*_k_*_,…_ 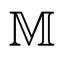 *_N,k_*} is a partition of 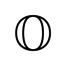 at time *k* subject to *μ_i,k_* ∈ 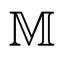 *_i,k_*, such as the Voronoi partition. The introduction of the partition is for avoidance of collision between UAVs and ease of dealing with the overlapped sensing regions between neighboring agents, which will be discussed later. Since *p_i,k_* ⩾ *q_i,k_*, 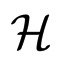 (*μ_k_*) is always nonnegative and 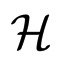 (*μ_k_*) = 0 if *h_i,k_* ⩾ *h̄*. Denoting by *∂*(•) the boundary of the corresponding region and *n*_∂(•)_ (*r*) the outward pointing normal vector of the boundary *∂* (•) at point *r*, we can compute the gradient of 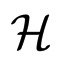 (*μ_k_*) as follows.

### Theorem 2

*The gradient of the cost function* 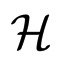 (*μ_k_*) *with respect to μ_i,k_* (*h_i,k_* < *h̄*) *is given by*

(18) $$\begin{array}{ll} \frac{\partial H(\mu_{k})}{\partial c_{i,k}} & =(p_{i,k}-q_{i,k})\int_{{\text{S}}_{i}^{2}}\phi_{k}(r)n_{{\text{S}}_{i}^{2}}(r)dr\\ \frac{\partial H(\mu_{k})}{\partial h_{i,k}} & =(f_{1}^{\prime }(h_{i,k})-f_{2}^{\prime }(h_{i,k}))\int_{{\text{S}}_{i}^{1}}\phi_{k}(r)dr+(p_{i,k}-q_{i,k})\tan\varphi \int_{{\text{S}}_{i}^{2}}\phi_{k}(r)dr \end{array}$$

*for h_i,k_* ∈ (*h̲, h̄*), *and*

(19) $$\begin{array}{ll} \frac{\partial H(\mu_{k})}{\partial c_{i,k}} & =(\overset{⌣}{p}-\hat{q})\int_{{\text{S}}_{i}^{2}}\phi_{k}(r)n_{{\text{S}}_{i}^{2}}(r)dr\\ \frac{\partial H(\mu_{k})}{\partial h_{i,k}} & =(\overset{⌣}{p}-\hat{q})\tan\varphi \int_{{\text{S}}_{i}^{2}}\phi_{k}(r)dr \end{array}$$

*for h_i,k_* ∈ (0, *h̲*), *where c*_*i*,0_ ∈ (0, *h̄*), ${\text{S}}_{i}^{1}={\text{M}}_{i,k}\cap {\text{C}}_{i,k}$, ${\text{S}}_{i}^{2}={\text{M}}_{i,k}\cap \partial \left({\text{C}}_{i,k}\right)$.

### Proof

See Appendix B.

Following Theorem 2, a gradient-based control law is given by

(20) $$u_{i,k}={K_{u}\frac{\partial H(\mu_{k})}{\partial \mu_{i,k}}|}_{\mu_{k}=\mu_{k-1}}$$

where *K_u_* is a positive gain parameter. A larger *K_u_* may lead to faster convergence of to the sub-optimal configuration, but may also cause larger convergence error or oscillation around the settle points due to the discrete-time control. In real system implementations, users should choose the parameter by trading off the two performance indices.

### Remark 4

*Note that the control input is always upper bounded in real systems, i.e.*, ‖*u_i,k_*‖ ⩽ *u_max_ for some positive number u_max_ and the height of each agent is also bounded by h_i,k_* < *h̄ to have meaningful detections. Moreover, to avoid collision when neighboring agents are at the same altitude, the motion of each agent should be constrained by c_i,k_*_+1_ ∈ 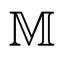 *_i,k_. Therefore, the control law* (20) *is modified as follows to be adapted to the constraints:*

(21) $$u_{i,k}={\lambda_{i,k}K_{u}\frac{\partial H(\mu_{k})}{\partial \mu_{i,k}}|}_{\mu_{k}=\mu_{k-1}}$$

*where λ_i,k_ is a scaling factor defined by*

(22) $$\begin{array}{l} \lambda_{i,k}=\underset{0⩽\lambda ⩽1}{\mathrm{argmax}}\left\|\lambda K_{u}\frac{\partial H(\mu_{k})}{\partial \mu_{i,k}}\right\|\\ \text{s.t.}\left\|\lambda K_{u}\frac{\partial H(\mu_{k})}{\partial \mu_{i,k}}\right\|⩽u_{\text{max}}\\ c_{i,k-1}+\lambda K_{u}\frac{\partial H(\mu_{k})}{\partial c_{i,k}}\in {\text{M}}_{i,k-1}\backslash \Lambda_{i,k-1}\\ h_{i,k-1}+\lambda K_{u}\frac{\partial H(\mu_{k})}{\partial h_{i,k}}⩽\bar{h}-\epsilon_{2} \end{array}$$

Λ*_i,k_ is a buffer region enclosing the border of* 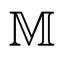 *_i,k_ defined as follows:*

(23) $$\Lambda_{i,k}=\left\{r\in {\text{M}}_{i,k}:\min\left(\epsilon_{1},\underset{s\in \partial {\text{M}}_{i,k}}{\min}\left\|c_{i,k}-s\right\|\right)<\underset{s\in \partial {\text{M}}_{i,k}}{\min}\left\|r-s\right\|\right\}$$

*where* ∊_1_, ∊_2_ > 0 *are given parameters for limiting the width of the buffer region and the height of each agent respectively*.

Generally, it is favored that UAVs stay longer in the region with less gathered information to take more measurements. Thus, we define the weighting function *ϕ_i,k_* (*g*) as a function of the gathered information ‖*Q_i,g,k_*_−1_‖ for each cell, *i.e.*:

(24) $$\phi_{i,k}(g)=e^{-K_{\phi }\left\|Q_{i,g,k-1}\right\|}$$

where *K_ϕ_* is a positive gain parameter. By this model, cells with less gathered information are given higher weights for detection. There is no specific rule for choosing the optimal *K_ϕ_* since it only denotes the user's preference on the search priority for different cells. In general, *K_ϕ_* is only required not to be too large or too small in order to properly scale the weights of different cells. For example, we find that *K_ϕ_* = 2 is one of the many suitable settings in our simulation.

### Remark 5

*The partition* { 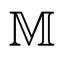
_1_*_,k_*, … 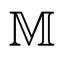 *_N,k_*} *can be static or time-varying. Partition is commonly used where each UAV only takes charge of one part of the whole surveillance region so that the whole searching task is shared by multiple agents. Users can predefine the task regions for each UAV or let the UAVs dynamically compute the partition following some rules. An example of the dynamic partition is the Voronoi partition which has been widely used in the distributed control* [13].

## 5. Simulation

### 5.1. Simulation Environment

**Figure 2.**
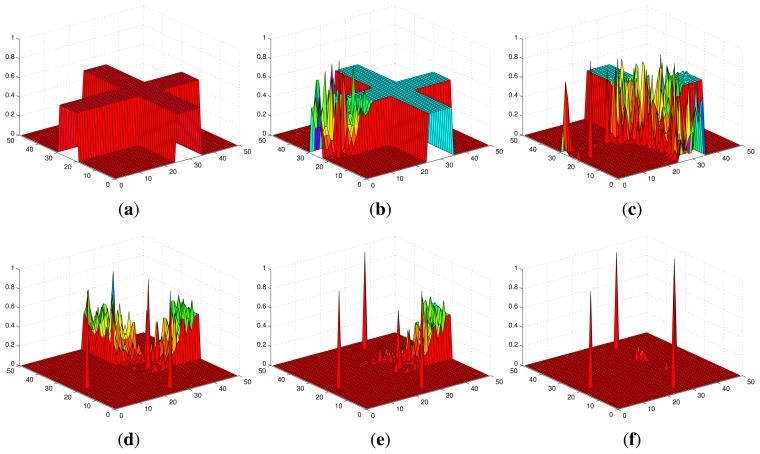
The convergence of the probability map of an agent in Scenario I. (**a**) *k* = 0 s; (**b**) *k* = 10 s; (**c**) *k* = 30 s; (**d**) *k* = 50 s; (**e**) *k* = 70 s; (**f**) *k* = 90 s.

We deploy multiple UAVs to search for four ground targets. The whole surveillance region is a square region of [0, 50] × [0, 50] m^2^ as shown in Figure 2a, within which lie two crossing roads denoted by 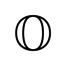 *_R_* ⊂ 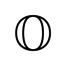. The four targets stay or move only on the roads and no target appears outside the roads in the surveillance region. At time *k*, each target *z* (*z* = 1,2,3,4) randomly moves to one of the cells in the set {*g* ∈ 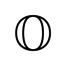 *_R_* : ‖*g* − **Tar***_z,k_*_−1_‖ ⩽ V_Tar_} where Tar*_z,k_*_−1_ is the cell it stays in at time *k* − 1 and V_Tar_ is the largest possible speed of target movement. Hence, *P* (*θ_g,k_* = 1|*θ_r,k_*−1 = 1) = 1/∑*_g_*_∈_**_
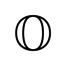
_***_R_* 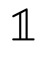
_{_*_g_*_∈_**_
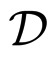
_***_r_*_}_ for *r* ∈ 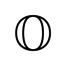 *_R_*, where 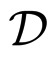 *_r_* = {*g* ∈ 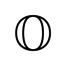 *_r_* : ‖*g* − *r*‖ ⩽ V_Tar_}. Initially, we set *Q_i,g_*_,0_ = 0 for all *i* and *g* within roads (*i.e.*, 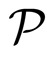 *_i,g,k_* = 0.5 for *g* ∈ 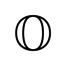 *r*), and *Q_i,g_*_,0_ to a fixed large value for *g* outside the roads (*i.e.*, 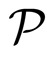 *_i_*,*_g,k_* ≈ 0 for *g*∉ 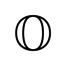 *_R_*). The detection probability function and the false alarm probability function are assumed to be *f*_1_ (*h_i,k_*) = *K*_1_*e*^−^*^K^*^2(^*^h^i,k*^−^*^h^*^)2^, and *f*_2_ (*h_i,k_*) = *K*_3_*e^K^*^4(^*^hi^*^,k−^*^h̲^*^)2^ respectively where *K*_1_, *K*_2_, *K*_3_,*K*_4_ are positive parameters satisfying the conditions in Equations (9) and (10). We test the proposed target search method in two scenarios. In Scenario I, all targets appear at *k* = 0 and keep stationary during the whole searching process, *i.e.*, V_Tar_ = 0 m/s. In Scenario II, we set V_Tar_ = 1 m/s to test the influence of target mobility on the convergence of probability maps. The four targets also appear at *k* = 0 but do not disappear during the search. In these two scenarios, we verify the effectiveness of the proposed target search method by deploying different number of UAVs and using different information decaying factors. The initial positions of UAVs are randomly selected within region [0,5]^3^ m^3^. The partition { 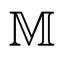
_1_,*_k_*,… 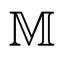 *_N,k_*} is generated by Voronoi partition. The communication range is set as *R_c_* = 20 m and the communication control protocol in [11] is applied for connectivity maintenance. A distributed *K*-connectivity maintenance algorithm has also been developed by the authors in [23] which can be applied in cooperative target search. Readers may refer to the references for more details on the communication protocols or maintenance algorithms. The cell size is fixed as 1 × 1 m^2^ Other key parameters are respectively set as *K_u_* = 0.3, *K_ϕ_* = 2, *q* = 0.1, *p̆* = 0.99, *qˆ* = 0.01, *h̄* = 10 m, *h̲* = 5 m, *α* = 0, *u_max_* = 2 m/s and *T* = 1 s.

Since the convergence of the individual probability map 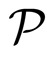 *_i_*,*_g,k_* of agent *i* implies that the weight *ϕ_i,k_* (*g*) defined by Equation (24) approaches 0 for each cell, we define the following average weight to evaluate the convergence performance of the whole network:

$$\phi_{k}=\frac{1}{NM_{R}}\overset{N}{\underset{i=1}{\text{∑}}}\underset{g\in {\text{O}}_{R}}{\text{∑}}\phi_{i,k}(g)$$

where *M_R_* denotes the total number of cells within the roads. It is easy to find that the initial value of *ϕ_k_*is $\phi_{0}=\frac{1}{NM_{R}}{\text{∑}}_{i=1}^{N}{\text{∑}}_{g\in {\text{O}}_{R}}e^{-K_{\phi }\left\|Q_{i,g,0}\right\|}=1$. In the simulations, we compare the results of *ϕ_k_* with different system parameters. The results are averaged from 200 Monte Carlo simulations.

### 5.2. Simulation Results

**Figure 3.**
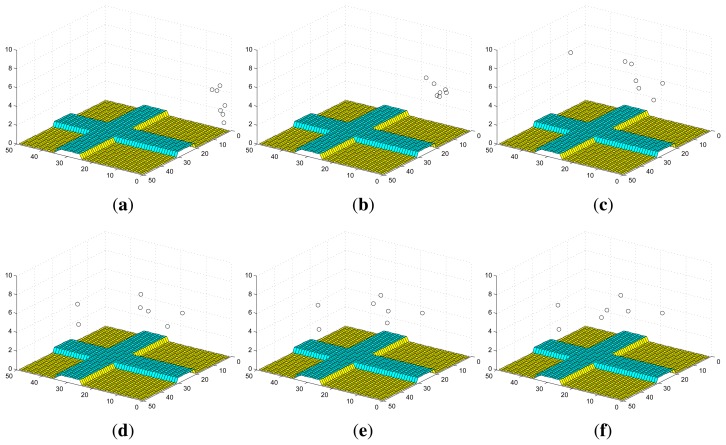
Snapshots of UAVs in Scenario I. (**a**) *k* = 0 s; (**b**) *k* = 10 s; (**c**) *k* = 30 s; (**d**) *k* = 50 s; (**e**) *k* = 70 s; (**f**) *k* = 90 s.

**Figure 4.**
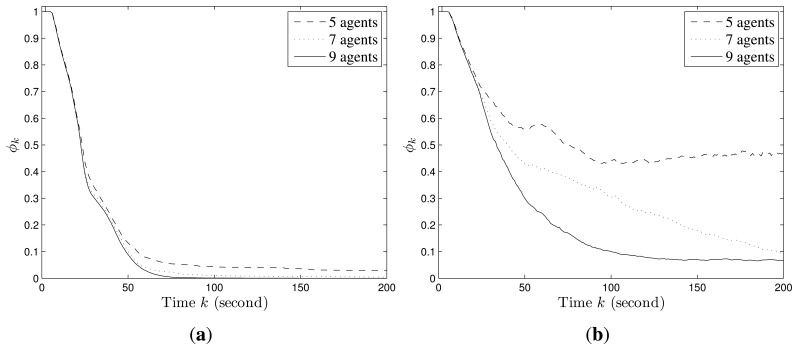
Weight average *ϕ_k_* by different number of agents. (**a**) Scenario I; (**b**) Scenario II.

Figure 2 shows an example of the convergence process of individual probability maps in Scenario I with stationary targets, where the probabilities converge to 1 for the cells within which targets truly exist and 0 for the cells within which no target exists. The snapshots of UAVs in Scenario I are shown in Figure 3. Additionally, *ϕ_k_* finally converges to 0 and the more agents are deployed, the faster it converges as shown in Figure 4a.

**Figure 5.**
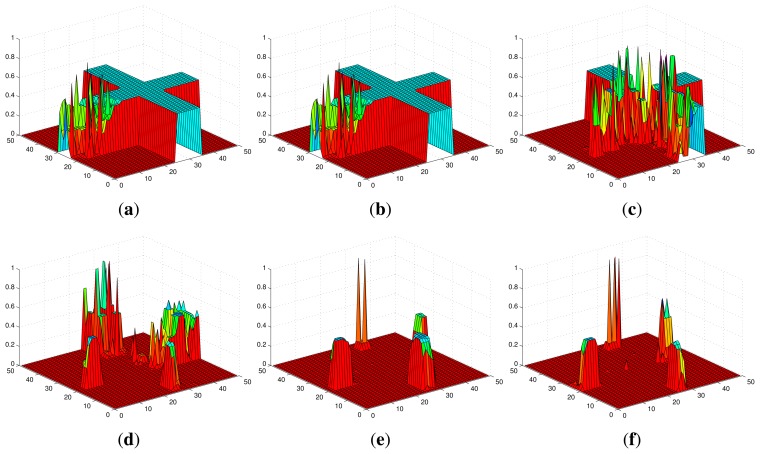
The convergence of the probability map of an agent in Scenario II. (**a**) *k* = 0 s; (**b**) *k* = 10 s; (**c**) *k* = 30 s; (**d**) *k* = 50 s; (**e**) *k* = 70 s; (**f**) *k* = 90 s.

**Figure 6.**
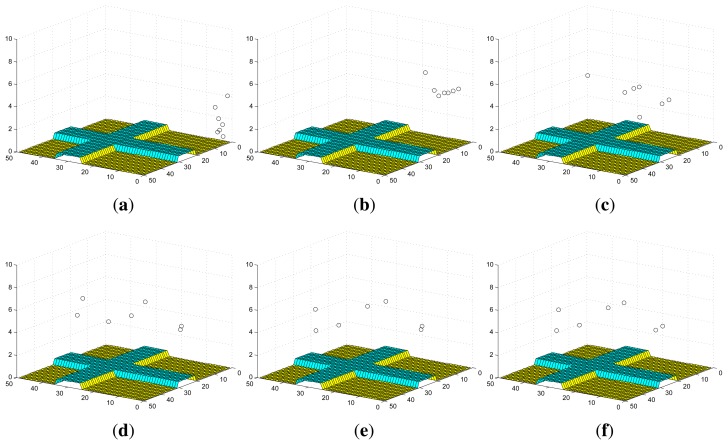
Snapshots of UAVs in Scenario II. (**a**) *k* = 0 s; (**b**) *k* = 10 s; (**c**) *k* = 30 s; (**d**) *k* = 50 s; (**e**) *k* = 70 s; (**f**) *k* = 90 s.

The convergence process of an individual probability map in Scenario II with mobile targets is shown in Figure 5, where the probabilities for the cells around targets may not converge to 0 as in Scenario I due to the random mobility of targets. However, we still can infer that there are four targets on the roads and have a rough estimation of their positions based on the envelopes of the final probability maps of UAVs. The snapshots of UAVs in Scenario II at according times are shown in Figure 6. In this case, *ϕ_k_* does not converge to 0 as shown in Figure 4b. However, a smaller *ϕ_k_* can be obtained with more agents deployed since the collective sensing area becomes larger. Compared with the results in Scenario I, the number of deployed agents has a greater impact on the convergence performance of probability maps in Scenario II with random target mobility. Hence, the algorithm is more robust with more UAVs deployed.

**Figure 7.**
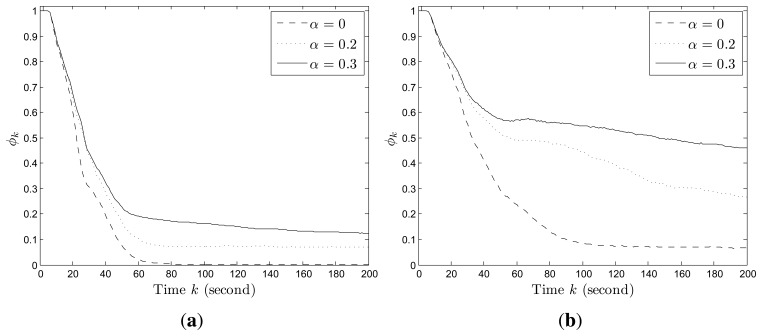
Weight average *ϕ_k_* by different information decaying factor. (**a**) Scenario I; (**b**) Scenario II.

In addition, we also test the impact of the information decaying factor on the convergence results. According to the simulation results (as shown in Figure 7), a larger decaying factor will lead to larger average uncertainty about the target existence in the whole region, because the accumulated information for each cell decays faster. In fact, the design purpose of the decaying factor is to let agents revisit each cell at certain frequency to update the latest information about target existence in the cell. Therefore, the tradeoff lies in that, a larger decaying factor leads to larger uncertainty, but makes the agents pay more attention to the cells with fewer observations. However, there is no quantitative means of choosing the decaying factor and users may find a proper one via simulation method.

## 6. Conclusions

In this paper, we studied the three-dimensional vision-based cooperative control and information fusion in target search by a group of UAVs with limited sensing and communication capabilities. First, heuristic detection probability and false alarm probability models were built which are related to the target discriminability of a camera and varies as a function of altitude. Then, we formulated the target search problem as a coverage optimization problem by balancing the coverage area and the detection performance. A generalized probability map updating model was proposed, by considering the information decay and transmission due to environmental changes such as the target movement. The simulation results showed that the proposed algorithms can make the individual probability maps of all agents converge to the same one which reflects the true environment when the targets are stationary. The influence of target mobility and the number of deployed UAVs on the convergence of probability maps has also been illustrated by simulation. Following this work, there is still a big potential area for future development and generalization of the proposed method. For example, the extension for detection by heterogenous sensors is an interesting topic since more types of information can be combined to improve the detection performance. More realistic environmental and system conditions that can affect the search results need to be considered such as the light intensity, block on the line of sight, camera with adjustable focus, asynchronous communication, *etc.*

## Appendix A. Proof of Theorem 1

First, we consider Case 1, where a target is present within cell *g*. Define the following augmented notations for the entire system:

$$\begin{array}{ll} {ϒ}_{g,k} & ≜{\left[Q_{1,g,k},Q_{2,g,k},\ldots ,Q_{N,g,k,}\right]}^{\text{T}}\\ \Phi_{g,k} & ≜{\left[\upsilon_{1,g,k},\upsilon_{2,g,k},\ldots ,\upsilon_{N,g,k,}\right]}^{\text{T}} \end{array}$$

Then, the updating rule (5) and (6) can be replaced by the following equation:

(25) $${ϒ}_{g,k}=\prod_{t=1}^{k}W_{t}{ϒ}_{g,0}+\overset{k}{\underset{l=1}{\text{∑}}}\prod_{t=l}^{k}W_{t}\Phi_{g,l}$$

Hence,

$$\text{E}\;\left[\overset{N}{\underset{i=1}{\text{∑}}}Q_{i,g,k}\right]=\overset{N}{\underset{i=1}{\text{∑}}}\text{E}\;\left[Q_{i,g,0}\right]+\overset{k}{\underset{t=1}{\text{∑}}}\overset{N}{\underset{t=1}{\text{∑}}}\underset{j\in N_{i,t}}{\text{∑}}\text{E}\;[\upsilon_{j,g,t}]$$

where

$$\text{E}\;\left[\upsilon_{j,g,t}\right]=\left[p_{j,t}\ln\frac{q_{j,t}}{p_{j,t}}+(1-p_{j,t})\ln\frac{1-q_{j,t}}{1-p_{j,t}}\right]1_{\left\{g\in {\mathbb{C}}_{j,t}\right\}}$$

and _{_*_g_*_∈ ℂ_*_j_*,*_t_*_}_ is the indicator function defined as:

$$1_{\left\{g\in {\mathbb{C}}_{j,t}\right\}}=\{\begin{array}{ll} 1 & \text{if}\;g\in {\mathbb{C}}_{j,t}\\ 0 & \text{otherwise} \end{array}$$

Since *Q_i,g_*,_0_, *υ_j_*_1_*_,g_*,*_η_*_1_ and *υ_j_*_2_*_,g_*,*_η_*_2_ are independent for *j*_1_ ≠ *j*_2_ or *η*_1_ ≠ *η*_2_, we can get the variance

$$\text{D}\;\left[\overset{N}{\underset{i=1}{\text{∑}}}Q_{i,g,k}\right]=\overset{N}{\underset{i=1}{\text{∑}}}\text{D}\;\left[Q_{i,g,0}\right]+\overset{k}{\underset{t=1}{\text{∑}}}\overset{N}{\underset{i=1}{\text{∑}}}\underset{j\in N_{i,t}}{\text{∑}}\text{D}\;\left[\upsilon_{i,g,l}\right]$$

where

$$D\left[\upsilon_{j,g,t}\right]=p_{j,t}(1-p_{j,t}){\left(\ln\frac{q_{j,t}}{p_{j,t}}-\ln\frac{1-q_{jt}}{1-p_{jt}}\right)}^{2}1_{\left\{g\in {\mathbb{C}}_{j,t}\right\}}$$

Considering that D [*υ_j,g,t_*] is a continuous function of *p_j,t_* and 1 > *p̌* ⩾ *p_j,t_* ⩾ 0.5 + *ε* > 0.5, 0 < *qˆ* ⩽ *q_j,t_* ⩽ 0.5 − *ε* < 0.5, there exists a constant real number $\sigma =2\sqrt{\overset{⌣}{p}(0.5-\varepsilon )}\ln\frac{0.5-\varepsilon }{0.5+\varepsilon }$ such that D [*υ_j,g_*,*_t_*] ⩽ *σ*^2^ for *g* ∈ ℂ*_j,t_*, which implies

$$\underset{m_{g,k}\to +\infty }{\lim}\overset{k}{\underset{t=1}{\text{∑}}}\overset{N}{\underset{i=1}{\text{∑}}}\underset{j\in N_{i,t}}{\text{∑}}\frac{\text{D}[\upsilon_{j,g,t}]}{m_{g,t}^{2}}⩽\underset{m_{g,k}\to +\infty }{\lim}\overset{m_{g,k}}{\underset{l=1}{\text{∑}}}\frac{N^{2}\sigma^{2}}{l^{2}}<\infty$$

According to the Kolmogorov Strong Law of Large Numbers, [24] we get

$$\begin{array}{cc} \begin{array}{cc} \frac{{\text{∑}}_{t=1}^{k}{\text{∑}}_{i=1}^{N}{\text{∑}}_{j\in N_{i,t}}\text{E}[\upsilon_{j,g,t}]}{m_{g,t}}\overset{a.s.}{\to }0, & as \end{array} & m_{g,t}\to +\infty \end{array}$$

Hence,

$$\begin{array}{ll} \frac{{\text{∑}}_{i=1}^{N}Q_{i,g,k}}{m_{g,k}} & =\frac{{\text{∑}}_{i=1}^{N}Q_{i,g,0}}{m_{g,k}}+\frac{{\text{∑}}_{t=1}^{k}{\text{∑}}_{i=1}^{N}{\text{∑}}_{j\in N_{i,t}}\text{E}\;[\upsilon_{j,g,t}]}{m_{g,t}}\\ & +\frac{{\text{∑}}_{t=1}^{k}{\text{∑}}_{i=1}^{N}{\text{∑}}_{j\in N_{i,t}}\left(\upsilon_{j,g,t}-\text{E}[\upsilon_{j,g,t}]\right)}{m_{g,t}}\\ & \overset{a.s.}{\to }\frac{{\text{∑}}_{t=1}^{k}{\text{∑}}_{i=1}^{N}{\text{∑}}_{j\in N_{i,t}}\text{E}[\upsilon_{j,g,t}]}{m_{g,t}},\text{as}\;m_{g,t}\to +\infty \end{array}$$

On the other hand, since *p_j,t_* > *q_j,t_*, it is straightforward to get

$$\begin{array}{ll} \frac{\partial \text{E}\;[\upsilon_{j,g,t}]}{\partial p_{j,t}} & =\ln\frac{p_{j,t}}{q_{j,t}}-\ln\frac{1-p_{j,t}}{1-q_{j,t}}<0\\ \frac{\partial \text{E}\;[\upsilon_{j,g,t}]}{\partial q_{j,t}} & =\ln\frac{p_{j,t}}{q_{j,t}}-\ln\frac{1-p_{j,t}}{1-q_{j,t}}>0 \end{array}$$

which implies

$$\text{E}\;[\upsilon_{j},g,t]⩽2\varepsilon \ln\frac{0.5-\varepsilon }{0.5+\varepsilon }<0$$

Hence,

$$\underset{m_{g,k}\to +\infty }{\lim\sup}\frac{{\text{∑}}_{i=1}^{N}Q_{i,g,k}}{m_{g,k}}⩽\underset{m_{g,k}\to +\infty }{\lim\sup}\frac{{\text{∑}}_{i=1}^{k}{\text{∑}}_{i=1}^{N}\text{E}[\upsilon_{i,g,t}]}{m_{g,k}}=\hat{p}\ln\frac{q}{\hat{p}}+(1-\hat{p})\ln\frac{1-q}{1-\hat{p}}<0$$

which implies ${\text{∑}}_{i=1}^{N}Q_{i,g,k}\underset{\to }{a.s.}-\infty$. Since the network is connected all the time, *Q_i,g,k_* for each agent *i* will almost surely converge to −∞ by implementing the average consensus protocol (6) (as shown in [11,25]).

Following the same procedure of the proof above, we can prove the conclusion of Case 2.

## Appendix B. Proof of Theorem 2

We first consider *h_i,k_* ∈ (*h̲, h̄*). From Equation (17), we get

$$H(\mu_{k})=\overset{N}{\underset{i=1}{\text{∑}}}\int_{{\text{M}}_{i,k}}\phi_{k}(r)(p_{i,k}-q_{i,k})1_{\left\{r\in {\mathbb{C}}_{i,k}\right\}}dr=\overset{N}{\underset{i=1}{\text{∑}}}\int_{{\text{M}}_{i,k}\cap {\mathbb{C}}_{i,k}}\phi_{k}(r)(p_{i,k}=q_{i,k})dr$$

Defining a set of agents for agent *i, i.e.*,  *_i,k_* = {*j* : *∂* (  *_j,k_*) ∩ ℂ*_i,k_* ≠ Ø}, it follows that

$$\begin{array}{ll} \frac{\partial H(\mu_{k})}{\partial \mu_{i,k}} & =\int_{{\text{S}}_{i}^{1}}\phi_{k}(r)\left(\frac{\partial p_{i,k}}{\partial \mu_{i,k}}-\frac{\partial q_{i,k}}{\partial \mu_{i,k}}\right)dr+\int_{{\text{S}}_{i}^{2}}\phi_{k}(r)(p_{i,k}-q_{i,k}){\frac{\partial r}{\partial \mu_{i,k}}}^{T}n{\text{S}}_{i}^{2}(r)dr\\ & +\underset{j\in {\overset{⌢}{N}}_{i,k}}{\text{∑}}\int_{{\text{S}}_{i}^{3}}\phi_{k}(r)(p_{i,k}-q_{i,k}){\frac{\partial r}{\partial \mu_{i,k}}}^{\text{T}}n{\text{S}}_{i}^{3}(r)dr\\ & +\underset{j\in {\overset{⌢}{N}}_{i,k}}{\text{∑}}\int_{{\text{S}}_{i}^{4}}\phi_{k}(r)(p_{i,k}-q_{i,k}){\frac{\partial r}{\partial \mu_{i,k}}}^{\text{T}}n{\text{S}}_{i}^{4}(r)dr \end{array}$$

where ${\text{S}}_{i}^{1}≜{\text{M}}_{i,k}\cap {\mathbb{C}}_{i,k}$, ${\text{S}}_{i}^{2}≜{\text{M}}_{i,k}\cap \partial ({\mathbb{C}}_{i,k})$, ${\text{S}}_{i,j}^{1}≜(\partial ({\text{M}}_{i,k})\backslash \partial (\text{O}))\cap {\mathbb{C}}_{i,k}$, ${\text{S}}_{i,j}^{2}≜(\partial ({\text{M}}_{j,k})\cap \partial (\text{O}))\cap {\mathbb{C}}_{i,k}$. For *r* ∈ ${\text{S}}_{i,j_{1}}^{1}\cap {\text{S}}_{i,j_{2}}^{1}$ (*j*_1_, *j*_2_ ∈  and *j*_1_ ≠ *j*_2_ we have $n_{{\text{S}}_{j_{1}}^{3}}(r)=-n_{{\text{S}}_{j_{2}}^{3}}(r)$. For *r* ∈ ${\text{S}}_{i,j}^{2}(j\in {\overset{⌢}{N}}_{i,k})$, we have ${\frac{\partial r}{\partial \mu_{i,k}}}^{\text{T}}=0$. Hence,

$$\begin{array}{ll} \frac{\partial H(\mu_{k})}{\partial \mu_{i,k}} & =\int_{{\text{S}}_{i}^{1}}\phi_{k}(r)\left(\frac{\partial p_{i,k}}{\partial \mu_{i,k}}-\frac{\partial q_{i,k}}{\partial \mu_{i,k}}\right)dr+\int_{{\text{S}}_{i}^{2}}\phi_{k}(r)(p_{i,k}-q_{i,k}){\frac{\partial r}{\partial \mu_{i,k}}}^{\text{T}}n{\text{S}}_{i}^{2}(r)dr \end{array}$$

From Equation (9) and according to the results of [15], we get

$$\begin{array}{lll} \frac{\partial p_{i,k}}{\partial c_{i,k}}=0, & \frac{\partial p_{i,k}}{\partial h_{i,k}}=f_{1}^{\prime }(h_{i},k), & \frac{\partial q_{i,k}}{\partial h_{i,k}}=f_{2}^{\prime }(h_{i,k}) \end{array}$$

and

$$\begin{array}{ll} {\frac{\partial r}{\partial c_{i,k}}}^{\text{T}}n{\text{S}}_{i}^{2}(r) & ={\frac{\partial r}{\partial c_{i,k}}}^{\text{T}}n{\text{S}}_{i}^{2}(r)\\ {\frac{\partial r}{\partial h_{i,k}}}^{\text{T}}n_{{\text{S}}_{i}^{2}}(r) & =\tan\varphi \end{array}$$

where *φ* is half of the angle width of the field of view of each agent (as shown in Figure 1b). Therefore, Equation (18) holds.

According to Equation (9) *p_i,k_* = *p̌* for *h_i,k_* ∈ (0, *h̲*). It is straightforward to get Equation (19) following the same procedure of proof as above.
